# Assessment of “Sugranineteen” Table Grape Maturation Using Destructive and Auto-Fluorescence Methods

**DOI:** 10.3390/foods11050663

**Published:** 2022-02-24

**Authors:** Najwane Hamie, Luigi Tarricone, Vincenzo Verrastro, Giuseppe Natrella, Michele Faccia, Giuseppe Gambacorta

**Affiliations:** 1 Department of Soil, Plant and Food Science, University of Bari Aldo Moro, 70126 Bari, Italy; najwane.hamie@uniba.it (N.H.); giuseppe.natrella@uniba.it (G.N.); michele.faccia@uniba.it (M.F.); 2 Research Centre for Viticulture and Enology, Council for Agricultural Research and Economics, CREA, 70010 Turi, Italy; luigi.tarricone@crea.gov.it; 3 Department of Mediterranean Organic Agriculture, Mediterranean Agronomic Institute of Bari, 70010 Valenzano, Italy; verrastro@iamb.it

**Keywords:** table grapes, optimal harvesting, technological maturity, phenolic maturity, non-destructive tools, Multiplex^®^ 3

## Abstract

The optimal harvesting of table grapes is commonly determined based on technological and phenolic indices analyzed over the course of its maturity. The classical techniques used for these analyses are destructive, time-consuming, and work for a limited number of samples that may not represent the heterogeneity of the vineyard. This study aimed to follow the ripening season of table grapes using non-destructive tools as a rapid and accurate alternative for destructive techniques. Grape samples were collected from a Sugranineteen vineyard during the ripening season to measure the basic maturity indices via wet chemistry, and total polyphenols, anthocyanins, and flavonoids were evaluated by spectrophotometry. Fluorescent readings were collected from intact clusters with a portable optical sensor (Multiplex^®^ 3, Force-A, France) that generates indices correlated to different maturity parameters. Results revealed strong relationships between the Multiplex^®^ indices ANTH_RG and FERARI and the skin anthocyanin content, with R^2^ values equal to 0.9613 and 0.8713, respectively. The NBI_R index was also related to total anthocyanins (R^2^ = 0.8032), while the SFR_R index was linked to the titratable acidity (R^2^ = 0.6186), the sugar content (R^2^ = 0.7954), and to the color index of red grapes (CIRG) (R^2^ = 0.7835). Results demonstrated that Multiplex^®^ 3 can be applied on intact clusters as an effective non-destructive tool for a rapid estimation of table grapes’ anthocyanin content.

## 1. Introduction

The maturity assessment of table grapes (*Vitis vinifera* L.) is necessary to determine the optimal harvesting time, which is a critical point for the postharvest handling period. Both destructive and non-destructive methods are applied in viticulture for crop monitoring and evaluation [1]. A common practice is to harvest the grapes based on technological and phenolic maturity indices that are measured using destructive laboratory analyses [2]. The technological maturity mainly includes the measurement of sugar content, titratable acidity, and the pH value of grape juice. The phenolic maturity reflects the ripeness of berry skin, pulp, and seeds, considering their phenolic compositions, and is expressed either as total polyphenols or skin anthocyanin content [3,4]. The total phenolic compounds are usually extracted from grape skins and estimated via spectrophotometry methods, such as the Folin–Ciocalteu assay [5]. The detailed profile of anthocyanins that are responsible for the red color in mature berries [6] is commonly identified by high pressure liquid chromatography (HPLC) [7]. Headspace solid phase microextraction (HS-SPME) followed by gas chromatography mass spectrometry (GC-MS) is applied for the analysis and quantification of some polyphenols in wine and grapes [8].

Although effective and precise, all these techniques are expensive, destructive, time-consuming, and consider a limited number of samples [9,10]. These problems demonstrate the limitations of laboratory analyses to properly estimate the grape status in the vineyard and to reflect the spatial and temporal heterogeneity of grapes throughout the maturation period. Consequently, researchers are increasingly oriented toward non-destructive techniques which are fast, accurate, and enable real-time analyses of fruit quality and maturity for a large number of samples [11,12,13]. Particularly, the optical methods were advantageously used for precision viticulture to solve the problem of grape heterogeneity in vineyards [12,14,15]. Among the most recent optical techniques, a method based on fruit auto-fluorescence has been successfully used for the monitoring of grape maturity [16,17]. The fluorescence indices were proved to reflect the epidermal phenolic content in wine grape leaves and skin anthocyanin content in grape berries [16,18,19]. Multiplex^®^ (FORCE–A, Orsay, France) is a commercial hand-held optical sensor that applies the chlorophyll fluorescence screening technique [15]. Several successful applications of Multiplex^®^ sensor were reported for the non-destructive determination of grape anthocyanins, the assessment of the spatial variability of grape color in the vineyard, and the ripening evaluation of different wine grape cultivars [12,17,20,21].

While most studies were applied to wine grapes, the present investigation aimed to assess the ripening of red table grapes using both destructive and non-destructive fluorescence-based methods. In practice, the objective was to evaluate the feasibility of using the fluorescence-based method to measure skin anthocyanin content along with maturity and to study the possible relationship between laboratory results and in-site fluorescence readings collected by Multiplex^®^ 3 from intact clusters.

## 2. Materials and Methods

### 2.1. Plant Material

The experiment was carried out during the ripening season of 2019 on *Vitis vinifera* L. cv. Sugranineteen (Scarlotta Seedless^®^ brand) grown in a commercial organic vineyard (Azienda Agricola Romanazzi S.r.l., Castellaneta Marina, Taranto, Italy) in Castellaneta Marina, south Italy. Scarlotta Seedless is a late-season seedless cultivar characterized by sweet, crisp, red- to dark-red-colored and oval-shaped berries. Grapevines were grafted onto a 1103 Paulsen rootstock, spaced 2.5 × 2.5 m, and trained to the Y-shaped trellis system and covered by plastic film to protect canopy and clusters from hail, wind, and rainfall. The plastic film was characterized by a high solar total transmissivity coefficient (83.7%), while the photosynthetically active radiation (PAR) total transmissivity coefficients was 81.8% and the long wave infrared (LWIR) transmissivity coefficient was 53.6%. The growing techniques were implemented according to the viticulture practices of organic table grapes in Italy, and the harvest time was determined according to the commercial maturity specifications set by the parent company Sun World International, LCC, Bakersfield, CA, USA.

### 2.2. Grape Sampling and In-Field Measurements

The monitoring of grape ripening was performed weekly from the onset of veraison on 1 August 2019 (day of the year, DOY = 213) until the harvest time on 7 October 2019 (DOY = 280). The experimental site was divided into two blocks of different irrigation systems: Scarlotta Block 1, SB1 (farmer irrigation) and Scarlotta Block 2, SB2 (controlled irrigation by an Internet of Things sensor-based system). Each block constituted 98 vines planted into 7 adjacent rows. At each sampling time, 3 berries per vine (294 berries per block) were removed from different parts of the clusters free of visible damages, with their pedicel still attached. In parallel, 2 fluorescence readings (196 measurements per block) were collected by Multiplex^®^ 3 from clusters attached to both sides of the grapevine (Section 2.3). Collected berries were stored at −20 °C until total polyphenols and skin anthocyanins extraction and quantification. Additionally, another 10 clusters were randomly collected 5 times during maturation to conduct the basic wet chemistry analysis and decide the optimal harvesting day (Section 2.4).

### 2.3. Optical Sensor and Indices

The fluorescence measurements of grapes were collected by the hand-held optical sensor Multiplex^®^ (FORCE-A, Orsay, France), which is described in detail elsewhere [17]. In this study, we used the version Multiplex^®^ 3 (MP3) that generates 12 signals produced by the combination of different excitation light emitting diodes (LED), i.e., UV and red–blue–green (RGB), and three photodiode detectors for fluorescence recordings, i.e., yellow (YF), red (RF), and far red (FRF). Real-time measurements were acquired directly in the vineyard on intact clusters. Among the parameters that MP3 can measure, this study focuses on the measurement of skin anthocyanins using 2 indices: ANTH_RG, which is based on the chlorophyll fluorescence excited with red (R) and green (G) lights, and FERARI (fluorescence excitation ratio anthocyanin relative index), which is based on the far-red chlorophyll fluorescence under red excitation [12,17]. The other studied indices include the FLAV index, which is proportional to skin flavonols, the simple fluorescent ratio (SFR_R), which is related to the chlorophyll content in leaves and berries, and the nitrogen balance index (NBI_R), which accounts for both epidermal phenolics and chlorophyll contents [22,23,24,25].

The mathematical expressions of the used fluorescence indices are defined as:

ANTH_RG = log (FRF_R/FRF_G),(1)

FERARI = log (5000/FRF_R),(2)

FLAV = log (FRF_R/FRF_UV),(3)

SFR_R = FRF_R/RF_R,(4)

NBI_R = FRF_UV/FRF_R,(5)

where FRF_R, FRF_G, and FRF_UV are far-red fluorescence under red, green, and UV excitation, respectively. RF_R is red fluorescence under red excitation. Mx fluorescence signals were corrected for residual electronic offsets and normalized to a fluorescence standard (blue plastic foil, FORCE-A, Orsay, France).

### 2.4. Chemical Analysis

The technological maturity of Sugranineteen was monitored starting from DOY = 246 to decide the optimal harvesting date. Three replicates of grape juice were extracted from 20 berries per replicate and then filtered after centrifugation (10 °C, 3000× *g*, 15 min). Total soluble solids (TSS) were determined using a DBR 95 digital refractometer (XS Instruments, Carpi, Italy) and the pH was measured with a pH meter (Eutech Instruments, Breda, The Netherlands, XS pH 2700). The titratable acidity (TA) was determined by titrating the juice with 0.1 N NaOH to an endpoint of 7.0 pH, and results are expressed as g/L tartaric acid equivalent. Berry color was collected from 294 berries per block using a portable spectrophotometer (CM–700d, Konica Minolta, Inc., Tokio, Japan). Results are expressed as a color index of red grapes (CIRG), calculated from the CIELAB color coordinates, i.e., hue angle (h), lightness (L*), and chroma (C*), and defined as ((180 − h)/(L* + C*)) [26]. This index enables an objective color evaluation of red grape cultivars at different ripening stages [27].

### 2.5. Analysis of Total Polyphenols, Anthocyanins, and Flavonoids

The assessment of total polyphenols, anthocyanins, and flavonoids during ripening was performed on skin extracts. Nine berries of different color tones (fully, medium, and slightly colored) were weighed using an electronic balance (Gibertini, Milan, Italy) before the skins were peeled and infused in 25 mL of ethanol–chloride solution (70:30:1; C_2_H_5_OH:H_2_O:HCl) for 24 h in darkness. Extracts were then filtered through 0.45 μm filter papers and stored at −20 °C until spectrophotometric analyses. Total polyphenols were quantified using the Folin–Ciocalteu method on 1:10 diluted skin extracts with distilled water. In an Eppendorf tube, 100 μL of distilled water, 100 μL of diluted skin extract, and 100 μL of Folin–Ciocalteu reagent were homogenized and incubated for 5 min at room temperature. Another incubation for 90 min was performed after adding 500 μL of 10% of sodium carbonate to the mixture, and the absorbance was then recorded at 750 nm using the DU^®^ 800 UV/Vis spectrophotometer (Beckman Coulter, Brea, CA, USA). The quantification of total anthocyanins and flavonoids was performed on 1:25 diluted skin extracts with the ethanol–chloride solution. In this case, the absorbance was determined within the UV-Vis spectrum range of 230–700 nm, and peaks were identified using the graphical method [28]. The obtained results of different parameters were expressed as mg/kg of grape berries (skin and flesh).

### 2.6. Antioxidant Activity

The antioxidant activity of Sugranineteen was assessed with ABTS and DPPH assays on grape juice obtained from nine weighed berries per sample and subjected to centrifugation (8000 RPM for 5 min at 10 °C) and filtration using 0.45 µm filters. The ABTS (2,2′-azino-bis(3-ethylbenzothiazoline-6-sulfonic acid)) assay was carried out according to a method reported elsewhere, with minor modifications [29]. This method is based on the ability of antioxidants to scavenge the ABTS^+^. The stock solution was obtained by reacting 7 mM of ABTS solution (0.0960 g/25 mL H_2_O) with 400 µL of potassium persulphate (K_2_S_2_O_8_) for 16 h at room temperature and in darkness. An aqueous solution of 100 mM ABTS^+^ was then prepared and diluted with water to an absorbance of 0.80 ± 0.005 at 734 nm. The absorbance of 950 µL of diluted ABTS^+^ solution added to 50 µL of filtered juice was then recorded at 734 nm after a sharp 8 min of incubation at room temperature and in darkness.

The DPPH (2,2-diphenyl-1-picrylhydrazyl) assay was performed based on a pre-explained method, with small adjustments [30]. A mother solution of DPPH 0.08 mM was produced by solubilizing 0.0031 g of DPPH powder in 100 mL of ethanol. The solution of DPPH was diluted with ethanol to an absorbance of 0.80 ± 0.003 at 517 nm to perform the spectrophotometric analysis. A mixture of 50 µL of filtered juice and 950 µL of diluted DPPH solution was incubated for 30 min at room temperature and in darkness to record the absorbance of the mixture at 517 nm. The obtained results of both assays are expressed as µM Trolox equivalent antioxidant capacity (TEAC)/g of grapes.

### 2.7. HPLC-DAD Anthocyanin Analysis

The anthocyanin composition of grape skins extracts was determined using an HPLC Waters 600 E device (Waters Inc., Milford, MA, USA) equipped with a quaternary pump, a photodiode array detector, and an injection valve with a 20 μL loop [31]. Skin extracts were filtered through 0.45 μm nylon membrane and diluted 1:2 with 10% formic acid. Samples were then injected into a NovaPack C18 (150 × 3.9 mm, 4 μm particle size, Waters Inc.) column maintained at 30 °C and eluted at a flow rate of 1 mL/min with 10% formic acid (solvent A) and acetonitrile (solvent B). The gradient program of solvent A was the following: 0–10 min 95%, 10–20 min 87%, 20–30 min 85%, 30–45 min 75%, and 45–50 min 95%. Anthocyanins were detected at 520 nm and quantitative analysis was performed according to an external standard method based on a calibration curve obtained by injecting different concentrations of malvidin-3-O-glucoside solutions (R^2^ = 0.9991). Anthocyanin compounds were identified by comparing the elution pattern and data reported in previous studies [32,33,34]. The results were expressed as mg/kg of malvidin-3-O-glucoside equivalents in grape berries.

### 2.8. Statistical Analysis

All destructive determinations were made in triplicate. Data processing and statistical analysis were carried out on Microsoft^®^ Excel and Minitab 20.3 (Minitab, LLC, State College, PA, USA) software. A one-way analysis of variance (ANOVA) with Tukey’s honest significant difference (HSD) test were performed for each studied parameter at a *p*-value ≤ 0.05.

## 3. Results

### 3.1. Analysis of Ripeness

The technological maturity of grapes was monitored starting from 3 September (DOY = 246) until harvest (Table 1). The pH of grape juice fluctuated with slight differences among blocks and maturation days and averaged 3.47 for SB1 and 3.61 for SB2 at harvest. The sugar content had an increasing trend, while the acidity was decreasing during maturation and reached at harvest an average TSS of 17.9 °Brix and an acidity value of 4.86 g/L with no significant differences among the blocks.

The CIRG averaged at harvest 5.09 and 5.32 for SB1 and SB2, respectively, reflecting the red color of the berries. The harvest was conducted on 7 October (DOY = 280) according to the measured parameters and the commercial maturity standards of Sugranineteen.

### 3.2. Polyphenols and Antioxidant Acitivy

The total amount of polyphenols, flavonoids, and anthocyanins measured using spectrophotometry was expressed as mg/kg of berry FW (Table 2). Total polyphenols fluctuated in a constant range during the ripening season between 586 and 996 mg/kg. Both blocks had almost the same trend, with slightly higher amounts of polyphenols for SB2, which showed an average concentration at harvest of 851 mg/kg compared to 738 mg/kg in SB1. Total flavonoids followed the same trend, as total polyphenols and fluctuated within a constant range until harvest. Flavonoids had significantly higher concentrations in SB1 during maturation, but at harvest, the content was slightly higher in SB2 (508.8 versus 468.7 mg/kg). In contrast, anthocyanins had an evident increasing trend over the entire maturation period, except for DOY 263, and had almost a similar trend among blocks, with mostly higher values for SB2. At harvest, SB2 reached an average value of 145 mg/kg, compared to 123 mg/kg for SB1.

Regarding the antioxidant activity, results were expressed as the µM Trolox equivalent antioxidant capacity (TEAC)/kg of grapes (Table 2). This was analyzed using two different assays, ABTS and DPPH. Despite the fluctuation of values, both methods revealed a similar increasing trend throughout maturation. ABTS expressed the antioxidant activity with higher concentration than DPPH, varying between 1.9 and 4.1 µM/kg. At harvest, no significant difference was detected between the two blocks, and the values averaged 3.4 and 3.8 µM/kg for SB1 and SB2, respectively. Results from DPPH followed a range of values between 0.9 and 2.5 µM/kg. At harvest, concentrations were also similar between the two blocks, averaging 2.2 µM/kg for SB1 and 2.5 µM/kg for SB2.

### 3.3. Anthocyanin Profile

**Figure 1:**
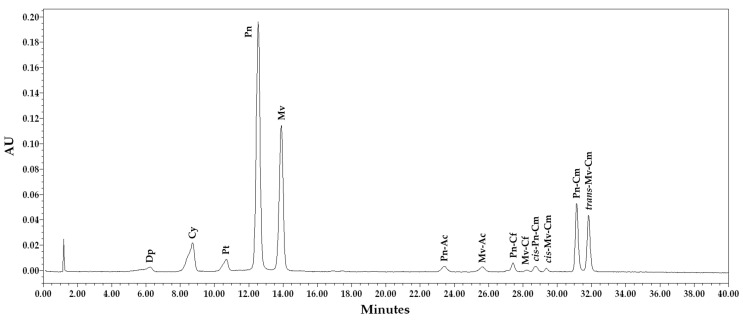
HPLC-DAD anthocyanin profile of SB2 Sugranineteen at DOY 280 (harvest). Dp, delphinidin-3-O-glucoside; Cy, cyanidin-3-O-glucoside; Pt, petunidin-3-O-glucoside; Pn, peonidin-3-O-glucoside; Mv, malvidin-3-O-glucoside; Pn-Ac, peonidin-3-O-acetyl-glucoside; Mv-Ac, malvidin-3-O-acetyl-glucoside; Pn-Cf, peonidin-3-O-caffeoyl-glucoside; Mv-Cf, malvidin-3-O-caffeoyl-glucoside; *cis*-Pn-Cm, *cis*-peonidin-3-O-coumaroyl-glucoside; *cis*-Mv-Cm, *cis*-malvidin-3-O-coumaroyl-glucoside; Pn-Cm, peonidin-3-O-coumaroyl-glucoside; *trans*-Mv-Cm, *trans*-malvidin-3-O-coumaroyl-glucoside.

A total of 13 anthocyanin compounds were identified and quantified using HPLC-DAD, as shown in Figure 1, corresponding to the SB2 sample at harvest time. The anthocyanin compositions of grape skin extracts are reported in Table 3. The most abundant anthocyanins at harvest were peonidin-3-O-glucoside (37.5 and 42.4 mg/kg) and malvidin-3-O-glucoside (17.4 and 24.7 mg/kg), followed by cyanidin-3-O-glucoside (14.9 and 8.8 mg/kg), peonidin-3-O-coumaroyl-glucoside (6.9 and 9.2 mg/kg), and *trans*-malvidin-3-O-coumaroyl-glucoside (3.9 and 7.3 mg/kg) in SB1 and SB2, respectively. All these compounds were significantly higher in SB2, except for cyanidin-3-O-glucoside.

Another observation is that the amounts of almost all the identified compounds were increasing with maturation, despite the fluctuation of values among the maturation days. At harvest, the total amount of anthocyanins reached 87.7 mg/kg for SB1, which is significantly lower than the content of SB2, which averaged 101.6 mg/kg of grapes.

### 3.4. Changes in Cluster Fluorescence during Maturation

**Figure 2:**
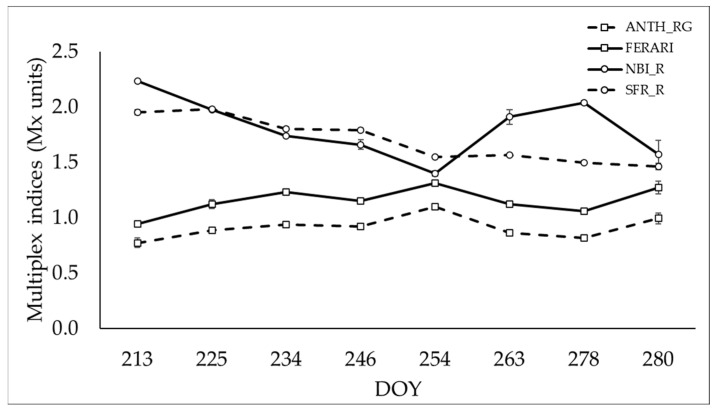
Changes in fluorescent indices (Mx units) of Sugranineteen clusters during ripening (day of the year, DOY).

The variation of Multiplex^®^ indices during the maturation period is shown in Figure 2. The collection of fluorescent readings was performed in parallel with berry sampling from DOY 213 to 280. The nitrogen balance index (NBI_R) that is correlated to the epidermal phenolics and chlorophyll decreased until DOY 254, then increased until DOY 278 and dropped again at harvest. The simple fluorescent ratio SFR_R that is correlated to the chlorophyll content in berries had a decreasing trend without fluctuation until DOY 280 (harvest time). In contrast, the indices correlated to the anthocyanin contents AHTH_RG and the FERARI index had a similar trend over the entire maturation period, with higher ranges for the former compared to the FERARI indices, and were increasing toward harvest time.

### 3.5. Relationship between Destructive and Fluorescent Measurements

**Figure 3:**
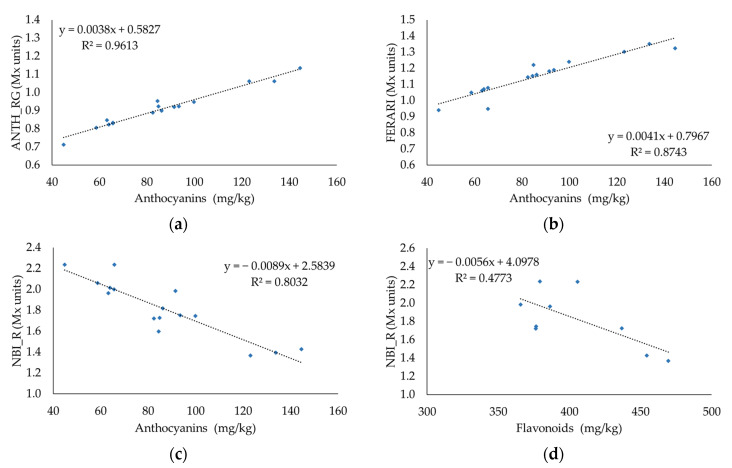

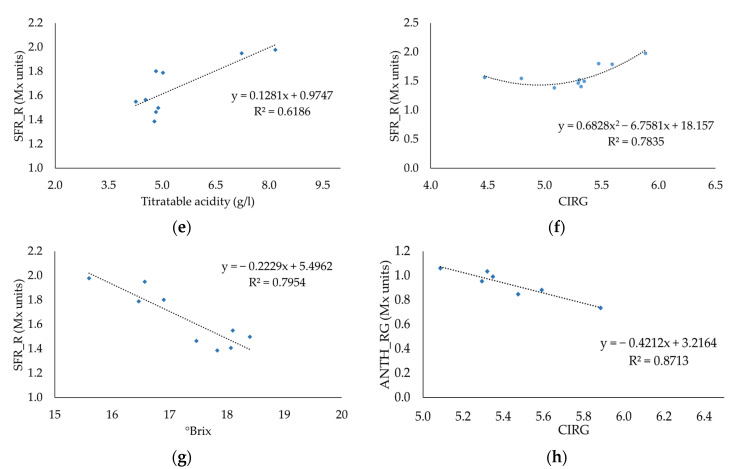
Regression models reflecting the relationships between different grape maturity parameters and the fluorescent measurements collected by Multiplex^®^ 3 along the maturation season: (**a**) relationship between ANTH_RG (Mx units) and anthocyanins (mg/kg) (n = 16); (**b**) relationship between FERARI (Mx units) and anthocyanins (mg/kg) (n = 16); (**c**) relationship between NBI_R (Mx units) and anthocyanins (mg/kg) (n = 16); (**d**) relationship between NBI_R (Mx units) and flavonoids (mg/kg) (n = 9); (**e**) relationship between SFR_R (Mx units) and titratable acidity (g/L) (n = 9); (**f**) relationship between SFR_R (Mx units) and CIRG (n = 10); (**g**) relationship between SFR_R (Mx units) and TSS (°Brix) (n = 9)); (**h**) relationship between ANTH_RG (Mx units) and CIRG (n = 7). Each dot represents the mean value of triplicates of laboratory analyses and the relative 196 in-vineyard-collected fluorescent measurements.

Regression model analyses were conducted to study the relationship between the different grape maturity parameters analyzed destructively and the fluorescent measurements collected by Multiplex^®^ 3 along the maturation season. Regression equations with the corresponding coefficient of determination (R^2^) are shown in Figure 3 and Table S1. The indices ANTH_RG and FERARI showed a significant relationship with skin anthocyanins (*p* < 0.001) and fitted a positive linear model, with R^2^ values equal to 0.9613 and 0.8743, respectively. The NBI_R index that is correlated to the epidermal phenolics and chlorophyll revealed significant negative linear relationships with anthocyanins (R^2^ = 0.8032; *p* < 0.001) and flavonoids (R^2^ = 0.4773; *p* = 0.039). Among the technological maturity parameters, TA, CIRG, and TSS were significantly related to the simple fluorescent index SFR_R that is associated with the chlorophyll content of berries, with R^2^ values equal to 0.6186 (*p* = 0.012), 0.7835 (*p* = 0.005), and 0.7954 (*p* = 0.001), respectively. The FLAV index that is correlated to flavonols did not show a significant relationship when compared with flavonoid content (Table S1).

## 4. Discussion

The optimal harvesting of Sugranineteen table grapes was assessed on DOY 280 based on the monitored technological maturity indices. The main indicator of the technological maturity was the TSS of grape juice that averaged at harvest 17.9 °Brix, with a corresponding low titratable acidity level (4.86 g/L). This sugar content was in line with the quality standards of Sun World International, LCC, that indicate a minimum TSS of 15.5 °Brix and the EU regulation (Reg. CE 1221/08) that claims a minimum of 14 °Brix for seedless table grapes. The skin color had a CIRG of 5.20 that reflects the red to dark-red color of the berries [35], which is the characteristic color of fully ripe Sugranineteen grapes. At harvest, all the measured indices were higher in SB2 (sensor-based controlled irrigation) than in SB1 (farmer irrigation), but no significant differences were detected except for pH (Table 1).

Regarding the phenolic maturity, total polyphenols and flavonoids fluctuated during the ripening period, while the anthocyanins had an increasing trend until harvest. The main reason behind this fluctuation, despite collecting the berries from the same grapevines at each sampling time, is the spatial and temporal heterogeneity of grape ripening in the vineyard [9,15]. This is a challenging characteristic of grapes in general because it affects the accurate monitoring of the maturity status, especially in large vineyards. The total anthocyanins reached at harvest was 144.6 mg/kg in SB2, and this was expected due to the high amounts of flavonoids (508.8 mg/kg) and total polyphenols (851.4 mg/kg). A study conducted on seedless table grapes from the Apulia region to quantify their total flavonoids revealed comparable results in 2007 for the black mid- and late-season cultivars Summer Royal and Autumn Royal, with 540 and 450 mg/kg, respectively. All the measured phenolic compounds were significantly higher in SB2 than in SB1 at harvest (Table 2), indicating a major maturation degree of grapes in controlled irrigation conditions and demonstrating the chemical composition (Table 1). The phenolic compounds are responsible for the organoleptic and qualitative characteristics of the fruit. Grapes with high levels of polyphenolic compounds appear redder or darker due to the presence of anthocyanins, which are colored flavonoid-type polyphenols mainly concentrated in the fruit epidermal tissues [36,37]. Except for the irrigation system, both blocks were grown using the same agricultural practices and environmental conditions. The sensor-based controlled irrigation system installed in SB2 provided an adequate amount of water to the grapevines, while the block SB1 was irrigated according to the farmer management. Following other studies, these results show the soil water content as a limiting factor affecting the overall quality of grape production [38]. The effect of different environmental factors on the accumulation of the phenolic compounds and their biosynthesis were previously demonstrated [39,40,41]. In general, the concentrations of polyphenols, anthocyanins, and flavonoids are influenced by the geographical site, climate conditions, soil fertility, cultivation practices, and pest management [42]. In addition to the environmental factors, the content of polyphenols varied widely between grape cultivars and growing seasons [42,43,44,45]. For instance, a study performed on Sugranineteen in 2014 showed a lower content of total polyphenols (451.4 mg/kg) and total anthocyanins (110.89 mg/kg) at harvest time [46] when compared to the reported results of season 2019 in Table 2.

In addition to being recognized as food sources of polyphenols [47], grapes are also known to be beneficial for human health, thanks to their antioxidant activity [48,49]. The present study has applied the two assays ABTS and DPPH to evaluate the antioxidant activity of Sugranineteen grapes. In parallel with total polyphenols, results fluctuated during the ripening season and were higher in SB2 than in SB1, although no statistical differences were detected. The antioxidant activity evaluated using the ABTS assay in SB2 reached 3.8 µM Trolox/kg at DOY 280, compared to 2.5 µM Trolox/kg when evaluated using the DPPH assay. Results showed that the antioxidant activity was better expressed by the ABTS method compared to DPPH, since the former revealed higher concentrations of antioxidant activity when testing the same samples along the maturation period. In fact, the ABTS assay is based on the ability of the sample to inhibit ABTS^+^, while the Trolox is used as a reference antioxidant standard, and the DPPH assay is a method that evaluates the ability of the sample to scavenge against the stable chromogenic radical DPPH.

The anthocyanin-3-O-glucosides and their acetyl, caffeoyl, and coumaroyl derivatives, eluted in the order Dp < Cy < Pt < Pn < Mv, were consistent with previous reports [43,50,51,52]. The most abundant anthocyanins throughout the maturation period were peonidin-3-O-glucoside and malvidin-3-O-glucoside, followed by cyanidin-3-O-glucoside, peonidin-3-O-coumaroyl-glucoside, and *trans*-malvidin-3-O-coumaroyl-glucoside. A similar profile of anthocyanin peaks was detected in the red Autumn Royal cultivar [43]. These results follow other studies, where it was found that the most abundant anthocyanins present in pink- and red-colored cultivars were peonidin-3-O-glucoside, whereas malvidin-3-O-glucoside, cyanidin-3-O-glucoside, and petunidin-3-O-glucoside forms were abundant in red-black cultivars [51,53]. This means that different proportions of individual anthocyanin compounds can affect the skin color of grapes [54].

Regarding the optical fluorescent measurements, the ANTH_RG and FERARI indices that are correlated to skin anthocyanins [17] were increasing toward harvest time. In parallel, the simple fluorescence index SFR_R and the nitrogen balance index NBI_R that are correlated to the chlorophyll content of the berries [17] were decreasing with the maturation time (Figure 2). These results reflect the normal variations that happened during the growing season, where the chlorophyll content that characterizes the green berries decreases and the anthocyanin content responsible for the color of berries rises with the ripening season. The trends of ANTH_RG, FERARI, SFR_R, and NBI_R during maturation were similar to the changes in the fluorescence ratios of Thompson Seedless grapes [55] and other wine grape varieties [21].

The comparison between destructive and non-destructive methods applied to assess the quality of table grapes was fruitful. Results revealed strong relationships among the technological and phenolic maturity indices measured in the laboratory and the fluorescent readings collected with the optical sensor Multiplex^®^ 3. This means that it has the ability to quantify the skin anthocyanin contents and other maturity indices of grape berries immediately and rapidly in-vineyard without collecting samples or using time-consuming technologies. Previous studies have described many calibrations of the Multiplex^®^ for anthocyanins estimation in wine grapes [12,17,20,24,56,57,58]. The most important relationships were found between the total anthocyanins estimated by spectrophotometry and the ANTH_RG and FEARI indices with R^2^ values equal to 0.9613 and 0.8743, respectively. These indices were also highly correlated to other wine grapes anthocyanins, such as Aleatico [12], Pinot Noir, Pinot Meunier, and Chardonnay [15]. In addition to these two indices, the NBI_R index that reflects the epidermal chlorophyll and anthocyanins was also correlated to total anthocyanins (R^2^ = 0.8032) and flavonoids, but with a low coefficient of determination (R^2^ = 0.4773). Regarding the technological maturity parameters, the simple fluorescent index SFR_R was correlated to the titratable acidity (R^2^ = 0.6186), CIRG (R^2^ = 0.7835), and total soluble solids (R^2^ = 0.7954); this was also detected in a previous study focused on wine grape cultivars [17]. Therefore, even though Multiplex^®^ 3 provides indices correlated to phenolic parameters, these results also revealed their good relationship with the technological indices of maturity. Only the FLAV index did not have a relationship with flavonoids, and the same results were found when it was compared to flavonols [12].

While almost all the studies were applied to wine grapes, these results revealed the new regression equations characteristic of Sugranineteen table grapes. The obtained models were generated from data collected from the veraison season (August 2019) until full maturity (October 2019); thus, they can be used for different stages of grape maturation. The relationship between the total anthocyanins evaluated through wet chemistry and the ANTH_RG index fitted a positive linear relationship. The same trend was also detected between the total anthocyanins and ANTH_RG index in Malvasia Rosa cultivar [21], while it was not the case of Tempranillo wine grape berries evaluated in the laboratory, which had a negative exponential model [20]. The prediction models depend on the cultivar and its morphological differences among grape varieties (size, weight, berry-skin thickness, and color) that may influence the fluorescent signals acquired by the Multiplex^®^ [59]. Another study suggests that the ANTH_RG index depends on the general anthocyanin profile and every single compound of the cultivar [58]. Moreover, the cultural practices and meteorological conditions that change among seasons can induce water stress status in the vines, thus affecting berry size and anthocyanin synthesis [12,60,61]. Therefore, more studies are required to characterize the ripening development of different table grape cultivars under different seasons and conditions when analyzed using Multiplex^®^.

## 5. Conclusions

The non-destructive assessment of grape maturity has recently become a promising technique in viticulture. The results demonstrated relationships between the different quality parameters analyzed destructively and the optical non-destructive fluorescent readings of Sugranineteen table grapes. The main finding was the regression equation developed between the ANTH_RG index from Multiplex^®^ 3 and the skin anthocyanin content, which helps to estimate this latter rapidly in-vineyard without damaging the plant material. While most of the previous studies were conducted on wine grape cultivars, this study assessed the whole maturity season of table grapes. This is a first step toward further promising studies to adjust the application of fluorescent techniques for the better estimation of the maturity status of different cultivars of table grape.

## Figures and Tables

**Table 1:** Changes in quality attributes of Sugranineteen grapes during ripening (mean values ± SD).

Quality Parameters	Sample	Maturation Time (DOY)				
		246	254	263	278	280 (Harvest)
pH	SB1	^A^ 3.35 ^d^ ± 0.01 *	^A^ 3.51 ^b^ ± 0.01	^A^ 3.56 ^a^ ± 0.01	^A^ 3.51 ^b^ ± 0.01	^B^ 3.47 ^c^ ± 0.01
	SB2	^A^ 3.34 ^c^ ± 0.01	^A^ 3.49 ^b^ ± 0.02	^B^ 3.44 ^b^ ± 0.02	^B^ 3.45 ^b^ ± 0.01	^A^ 3.61 ^a^ ± 0.01
TSS (°Brix)	SB1	^A^ 16.6 ^b^ ± 0.1	^A^ 16.9 ^b^ ± 0.2	^A^ 18.1 ^a^ ± 0.1	^A^ 18.4 ^a^ ± 0.2	^A^ 17.8 ^a^ ± 0.1
	SB2	^B^ 15.6 ^cd^ ± 0.3	^A^ 16.5 ^bc^ ± 0.5	^B^ 14.8 ^d^ ± 0.3	^B^ 17.5 ^ab^ ± 0.1	^A^ 18.1 ^a^ ± 0.2
Titratable acidity (g/L)	SB1	^B^ 7.23 ^a^ ± 0.58	^A^ 4.83 ^b^ ± 0.03	^B^ 4.26 ^c^ ± 0.15	^A^ 4.89 ^b^ ± 0.19	^A^ 4.78 ^b^ ± 0.08
	SB2	^A^ 8.18 ^a^ ± 0.09	^A^ 5.03 ^b^ ± 0.30	^A^ 4.54 ^b^ ± 0.04	^A^ 4.83 ^b^ ± 0.05	^A^ 4.94 ^b^ ± 0.10
CIRG	SB1	^B^ 5.30 ^a^ ± 0.07	^A^ 5.47 ^a^ ± 0.32	^A^ 4.80 ^b^ ± 0.14	^A^ 5.35 ^a^ ± 0.14	^A^ 5.09 ^ab^ ± 0.16
	SB2	^A^ 5.89 ^a^ ± 0.21	^A^ 5.59 ^ab^ ± 0.19	^B^ 4.47 ^c^ ± 0.08	^A^ 5.29 ^b^ ± 0.08	^A^ 5.32 ^b^ ± 0.20

* On the same row, means with different right superscripts differ significantly (*p* ≤ 0.05). Between the SB1 (farmer irrigation) and SB2 (sensor-based controlled irrigation) pair, means with different left superscripts differ significantly (*p* ≤ 0.05).

**Table 2:** Analyses of polyphenols and antioxidant activity of Sugranineteen grapes during ripening (mean values ± SD).

Parameters	Sample	Maturation Time (DOY)							
		213	225	234	246	254	263	278	280 (Harvest)
Total polyphenols (mg/kg)	SB1	^B^ 715.3 ^b^ ± 9.2 *	^B^ 911.7 ^a^ ± 56.6	^A^ 669.2 ^b^ ± 44.9	^A^ 846.9 ^ab^ ± 95.5	^A^ 746.5 ^b^ ± 33.9	^A^ 710.1 ^b^ ± 30.0	^A^ 813.8 ^ab^ ± 59.1	^B^ 737.5 ^b^ ± 54.9
	SB2	^A^ 819.9 ^b^ ± 40.7	^A^ 995.9 ^a^ ± 16.2	^A^ 684.0 ^c^ ± 16.2	^A^ 913.6 ^ab^ ± 89.0	^A^ 731.4 ^bc^ ± 54.6	^B^ 586.3 ^d^ ± 6.8	^A^ 785.6 ^b^ ± 36.1	^A^ 851.4 ^ab^ ± 43.4
Flavonoids (mg/kg)	SB1	^A^ 405.7 ^c^ ± 7.4	^A^ 542.6 ^a^ ± 35.1	^A^ 386.4 ^c^ ± 38.4	^A^ 480.8 ^ab^ ± 55.2	^A^ 436.8 ^b^ ± 9.3	^A^ 376.4 ^c^ ± 22.4	^A^ 469.4 ^b^ ± 36.0	^A^ 468.7 ^b^ ± 28.0
	SB2	^B^ 379.3 ^b^ ± 11.2	^B^ 485.0 ^a^ ± 13.7	^A^ 365.7 ^b^ ± 17.9	^A^ 473.7 ^a^ ± 20.8	^B^ 376.5 ^b^ ± 34.5	^B^ 289.8 ^c^ ± 1.7	^A^ 454.4 ^a^ ± 24.8	^A^ 508.8 ^a^ ± 33.0
Anthocyanins (mg/kg)	SB1	^B^ 44.8 ^e^ ± 7.4	^A^ 86.2 ^bc^ ± 7.5	^B^ 63.2 ^d^ ± 14.6	^A^ 84.9 ^bc^ ± 14.3	^A^ 82.4 ^c^ ± 1.6	^A^ 63.9 ^d^ ± 0.1	^B^ 93.4 ^b^ ± 6.4	^B^ 123.0 ^a^ ± 4.1
	SB2	^A^ 65.7 ^d^ ± 11.4	^B^ 65.6 ^d^ ± 9.7	^A^ 91.5 ^c^ ± 10.8	^A^ 99.9 ^c^ ± 1.6	^A^ 84.5 ^c^ ± 19.2	^B^ 58.6 ^d^ ± 2.1	^A^ 133.7 ^b^ ± 4.3	^A^ 144.6 ^a^ ± 1.7
Antioxidant activity									
ABTS (µM/g)	SB1	^A^ 3.6 ^a^ ± 0.1	^A^ 3.5 ^a^ ± 0.1	^A^ 2.7 ^b^ ± 0.2	^A^ 2.0 ^c^ ± 0.1	^A^ 2.9 ^b^ ± 0.2	^A^ 3.7 ^a^ ± 0.1	^A^ 4.1 ^a^ ± 0.6	^A^ 3.4 ^a^ ± 0.2
	SB2	^B^ 2.9 ^c^ ± 0.1	^A^ 3.3 ^b^ ± 0.1	^B^ 2.1 ^d^ ± 0.1	^A^ 2.1 ^d^ ± 0.1	^B^ 1.9 ^d^ ± 0.1	^B^ 3.1 ^bc^ ± 0.1	^A^ 3.7 ^a^ ± 0.2	^A^ 3.8 ^a^ ± 0.2
DPPH (µM/g)	SB1	^A^ 0.9 ^c^ ± 0.1	^A^ 1.2 ^c^ ± 0.2	^A^ 1.7 ^b^ ± 0.1	^A^ 1.6 ^b^ ± 0.1	^A^ 1.8 ^b^ ± 0.1	^A^ 2.0 ^ab^ ± 0.1	^A^ 2.4 ^a^ ± 0.4	^A^ 2.2 ^ab^ ± 0.3
	SB2	^A^ 1.1 ^d^ ± 0.1	^A^ 1.2 ^d^ ± 0.1	^B^ 1.4 ^cd^ ± 0.1	^A^ 1.6 ^c^ ± 0.1	^B^ 1.2 ^d^ ± 0.1	^A^ 2.0 ^b^ ± 0.1	^A^ 2.1 ^b^ ± 0.1	^A^ 2.5 ^a^ ± 0.1

* On the same row, means with different right superscripts differ significantly (*p* ≤ 0.05). Between the SB1 (farmer irrigation) and SB2 (sensor-based controlled irrigation) pair, means with different left superscripts differ significantly (*p* ≤ 0.05).

**Table 3:** Anthocyanin composition of Sugranineteen grapes during ripening (mg/kg, mean values ± SD).

Anthocyanins	Sample	Maturation Time (DOY)							
		213	225	234	246	254	263	278	280 (Harvest)
Dp	SB1	^B^ 0.5 ^d^ ± 0.2 *	^A^ 2.0 ^a^ ± 0.2	^B^ 1.0 ^c^ ± 0.1	^B^ 1.4 ^b^ ± 0.2	^A^ 1.7 ^ab^ ± 0.1	^A^ 0.7 ^cd^ ± 0.2	^B^ 0.3 ^d^ ± 0.1	^A^ 1.2 ^bc^ ± 0.1
	SB2	^A^ 1.4 ^c^ ± 0.1	^A^ 1.9 ^b^ ± 0.3	^A^ 1.7 ^bc^ ± 0.3	^A^ 2.7 ^a^ ± 0.4	^B^ 0.7 ^d^ ± 0.2	^A^ 0.5 ^d^ ± 0.2	^A^ 1.5 ^bc^ ± 0.5	^A^ 1.4 ^c^ ± 0.1
Cy	SB1	^B^ 2.2 ^d^ ± 0.4	^A^ 2.9 ^cd^ ± 0.4	^A^ 1.9 ^d^ ± 0.3	^A^ 2.6 ^cd^ ± 0.7	^A^ 3.1 ^c^ ± 0.4	^A^ 1.2 ^e^± 0.1	^A^ 10.6 ^b^ ± 0.1	^A^ 14.9 ^a^ ± 0.3
	SB2	^A^ 5.9 ^b^ ± 1.5	^A^ 2.5 ^c^ ± 0.2	^A^ 2.4 ^c^ ± 0.5	^A^ 2.2 ^c^ ± 0.1	^B^ 1.0 ^d^ ± 0.3	^B^ 0.7 ^d^ ± 0.1	^B^ 5.3 ^b^ ± 1.7	^B^ 8.8 ^a^ ± 0.6
Pt	SB1	^B^ 1.0 ^d^ ± 0.2	^A^ 2.5 ^a^ ± 0.2	^B^ 1.3 ^cd^ ± 0.2	^B^ 2.1 ^ab^ ± 0.2	^A^ 2.2 ^a^ ± 0.1	^A^ 1.6 ^c^ ± 0.1	^A^ 1.3 ^cd^ ± 0.2	^B^ 1.9 ^b^ ± 0.1
	SB2	^A^ 2.4 ^b^ ± 0.1	^A^ 2.3 ^b^ ± 0.3	^A^ 2.4 ^b^ ± 0.3	^A^ 3.5 ^a^ ± 0.5	^B^ 0.9 ^c^ ± 0.3	^B^ 0.3 ^d^ ± 0.2	^A^ 2.0 ^b^ ± 0.6	^A^ 2.5 ^b^ ± 0.1
Pn	SB1	^B^ 16.4 ^cd^ ± 3.9	^A^ 21.5 ^c^ ± 2.3	^A^ 21.5 ^c^ ± 4.5	^A^ 19.5 ^cd^ ± 5.0	^A^ 16.3 ^d^ ± 0.1	^A^ 11.4 ^e^ ± 0.1	^A^ 31.7 ^b^ ± 1.3	^B^ 37.5 ^a^ ± 0.5
	SB2	^A^ 34.2 ^b^ ± 3.3	^B^ 15.4 ^d^ ± 1.6	^A^ 26.9 ^bc^ ± 4.8	^A^ 16.7 ^d^ ± 0.9	^B^ 7.1 ^e^ ± 2.1	^A^ 10.0 ^e^ ± 2.1	^B^ 22.6 ^c^ ± 3.2	^A^ 42.4 ^a^ ± 0.5
Mv	SB1	^B^ 10.5 ^c^ ± 1.5	^A^ 23.0 ^a^ ± 1.3	^B^ 15.8 ^bc^ ± 3.7	^B^ 23.1 ^a^ ± 2.4	^A^ 21.0 ^ab^ ± 2.1	^A^ 15.6 ^bc^ ± 0.8	^A^ 11.5 ^c^ ± 1.8	^B^ 17.4 ^b^ ± 2.1
	SB2	^A^ 23.0 ^b^ ± 2.1	^A^ 19.7 ^bc^ ± 3.0	^A^ 24.1 ^b^ ± 2.9	^A^ 32.5 ^a^ ± 1.6	^B^ 8.4 ^c^ ± 2.5	^A^ 14.1 ^c^ ± 2.4	^A^ 15.8 ^c^ ± 5.0	^A^ 24.7 ^b^ ± 0.9
Pn-Ac	SB1	^A^ 0.2 ^c^ ± 0.1	^A^ 0.3 ^d^ ± 0.1	^A^ 0.4 ^c^ ± 0.1	^A^ 0.7 ^b^ ± 0.1	^A^ 0.8 ^b^ ± 0.1	^A^ 0.6 ^bc^ ± 0.1	^A^ 0.8 ^b^ ± 0.1	^A^ 1.1 ^a^ ± 0.1
	SB2	^A^ 0.7 ^b^ ± 0.3	^A^ 0.6 ^b^ ± 0.2	^A^ 0.5 ^c^ ± 0.1	^A^ 0.9 ^b^ ± 0.1	^B^ 0.4 ^c^ ± 0.1	^B^ 0.3 ^c^ ± 0.1	^A^ 0.9 ^ab^ ± 0.3	^A^ 1.2 ^a^ ± 0.1
Mv-Ac	SB1	^B^ 0.3 ^c^ ± 0.1	^A^ 0.8 ^ab^ ± 0.1	^B^ 0.5 ^bc^ ± 0.1	^B^ 0.8 ^ab^ ± 0.2	^A^ 0.9 ^a^ ± 0.1	^A^ 0.9 ^a^ ± 0.1	^B^ 0.4 ^ab^ ± 0.1	^B^ 0.6 ^b^ ± 0.1
	SB2	^A^ 0.8 ^bc^ ± 0.3	^A^ 0.6 ^c^ ± 0.1	^A^ 1.0 ^b^ ± 0.1	^A^ 1.5 ^a^ ± 0.1	^B^ 0.3 ^d^ ± 0.1	^A^ 0.7 ^c^ ± 0.1	^A^ 0.8 ^bc^ ± 0.2	^A^ 1.1 ^b^ ± 0.1
Pn-Cf	SB1	^B^ 0.1 ^e^ ± 0.1	^A^ 0.7 ^cd^ ± 0.1	^A^ 0.3 ^de^ ± 0.1	^A^ 0.5 ^d^ ± 0.1	^A^ 0.9 ^c^ ± 0.1	^A^ 0.5 ^cd^ ± 0.1	^A^ 1.3 ^b^ ± 0.1	^A^ 1.6 ^a^ ± 0.1
	SB2	^A^ 0.6 ^bc^ ± 0.3	^A^ 0.7 ^b^ ± 0.1	^A^ 0.4 ^c^ ± 0.1	^A^ 0.7 ^b^ ± 0.1	^B^ 0.3 ^b^ ± 0.1	^A^ 0.7 ^b^ ± 0.1	^A^ 1.0 ^ab^ ± 0.3	^B^ 1.2 ^a^ ± 0.1
Mv-Cf	SB1	^A^ 0.3 ^ab^ ± 0.1	nd	^A^ 0.1 ^b^ ± 0.1	^A^ 0.2 ^ab^ ± 0.1	nd	^A^ 0.4 ^a^ ± 0.1	nd	nd
	SB2	^A^ 0.3 ^b^ ± 0.1	nd	^A^ 0.2 ^b^ ± 0.1	^A^ 0.2 ^b^ ± 0.1	^A^ 0.1 ^b^ ± 0.1	^A^ 0.8 ^a^ ± 0.2	nd	^A^ 0.3 ^b^ ± 0.1
*Cis*-Pn-Cm	SB1	^B^ 0.2 ^b^ ± 0.1	^A^ 0.6 ^a^ ± 0.1	^A^ 0.3 ^b^ ± 0.1	^B^ 0.6 ^a^ ± 0.1	^A^ 0.6 ^a^ ± 0.1	^A^ 0.6 ^a^ ± 0.1	^A^ 0.6 ^a^ ± 0.1	^A^ 0.7 ^a^ ± 0.1
	SB2	^A^ 0.6 ^bc^ ± 0.2	^A^ 0.6 ^b^ ± 0.1	^A^ 0.5 ^bc^ ± 0.1	^A^ 1.0 ^a^ ± 0.1	^A^ 0.4 ^b^ ± 0.1	^B^ 0.3 ^c^ ± 0.1	^A^ 0.8 ^ab^ ± 0.2	^A^ 0.9 ^ab^ ± 0.1
*cis*-Mv-Cm	SB1	^A^ 0.1 ^a^ ± 0.1	^B^ 0.2 ^a^ ± 0.1	^A^ 0.2 ^a^ ± 0.1	^B^ 0.3 ^a^ ± 0.1	^A^ 0.3 ^a^ ± 0.1	^A^ 0.3 ^a^ ± 0.1	^A^ 0.1 ^a^ ± 0.1	^B^ 0.2 ^a^ ± 0.1
	SB2	^A^ 0.3 ^b^ ± 0.1	^A^ 0.3 ^b^ ± 0.1	^A^ 0.2 ^b^ ± 0.1	^A^ 0.6 ^a^ ± 0.1	^B^ 0.1 ^b^ ± 0.1	^A^ 0.2 ^bc^ ± 0.1	^A^ 0.3 ^b^ ± 0.1	^A^ 0.4 ^ab^ ± 0.1
Pn-Cm	SB1	^B^ 1.5 ^f^ ± 0.3	^A^ 2.9 ^e^ ± 0.2	^B^ 2.7 ^e^ ± 0.8	^B^ 3.7 ^cd^ ± 0.9	^A^ 4.5 ^c^ ± 0.1	^A^ 3.7 ^d^ ± 0.1	^A^ 6.0 ^b^ ± 0.1	^B^ 6.9 ^a^ ± 0.1
	SB2	^A^ 5.6 ^b^ ± 1.3	^A^ 2.9 ^c^ ± 0.4	^A^ 3.9 ^bc^ ± 0.6	^A^ 5.0 ^b^ ± 0.3	^B^ 2.5 ^c^ ± 0.7	^B^ 2.8 ^c^ ± 0.2	^A^ 5.7 ^b^ ± 1.8	^A^ 9.2 ^a^ ± 0.2
*trans*-Mv-Cm	SB1	^B^ 1.2 ^e^ ± 0.1	^A^ 3.9 ^c^ ± 0.2	^B^ 3.5 ^cd^ ± 0.9	^B^ 6.2 ^ab^ ± 0.9	^A^ 6.8 ^a^ ± 0.9	^A^ 5.5 ^b^ ± 0.1	^B^ 2.5 ^d^ ± 0.4	^B^ 3.9 ^c^ ± 0.7
	SB2	^A^ 4.7 ^cd^ ± 2.1	^A^ 3.8 ^cd^ ± 0.7	^A^ 5.4 ^c^ ± 0.5	^A^ 9.9 ^a^ ± 0.3	^B^ 2.9 ^d^ ± 0.9	^A^ 5.1 ^c^ ± 0.7	^A^ 5.3 ^c^ ± 1.5	^A^ 7.3 ^b^ ± 0.2
Total	SB1	^B^ 34.5 ^d^ ± 5.8	^A^ 61.5 ^bc^ ± 4.8	^A^ 49.4 ^cd^ ± 10.9	^B^ 61.6 ^bc^ ± 10.6	^A^ 59.0 ^c^ ± 2.7	^A^ 42.8 ^d^ ± 1.3	^A^ 67.0 ^b^ ± 4.0	^B^ 87.7 ^a^ ± 2.4
	SB2	^A^ 80.5 ^b^ ± 15.3	^A^ 51.3 ^c^ ± 7.0	^A^ 69.5 ^b^ ± 9.9	^A^ 77.3 ^b^ ± 4.5	^B^ 25.1 ^e^ ± 7.5	^B^ 36.6 ^d^ ± 0.9	^A^ 61.8 ^bc^ ± 19.7	^A^ 101.6 ^a^ ± 0.4

* On the same row, means with different right superscripts differ significantly (*p* ≤ 0.05). Between the SB1 (farmer irrigation) and SB2 (sensor-based controlled irrigation) pair, means with different left superscripts differ significantly (*p* ≤ 0.05). nd, not detected. For anthocyanins code, see the note to Figure 1.

## Data Availability

Not applicable.

## Supplementary Materials

The following supporting information can be downloaded at: https://www.mdpi.com/article/10.3390/foods11050663/s1, Table S1: Relationships between the Multiplex^®^ 3 ratios and the different quality parameters of Sugranineteen table grapes performed in this study. The ratios are described according to the manufacturer (Force A, Orsay, France).

## References

[1] Krstic M. (2003). Growing Quality Grapes to Winery Specification: Quality Measurement and Management Options for Grapegrowers.

[2] Nogales-Bueno J., Hernández-Hierro J.M., Rodríguez-Pulido F.J., Heredia F.J. (2014). Determination of technological maturity of grapes and total phenolic compounds of grape skins in red and white cultivars during ripening by near infrared hyperspectral image: A preliminary approach. Food Chem..

[3] Ferrer-Gallego R., Hernández-Hierro J.M., Rivas-Gonzalo J.C., Escribano-Bailón M.T. (2012). Influence of climatic conditions on the phenolic composition of *Vitis vinifera* L. cv. Graciano. Anal. Chim. Acta.

[4] Meléndez E., Ortiz M., Sarabia L., Íñiguez M., Puras P. (2013). Modelling phenolic and technological maturities of grapes by means of the multivariate relation between organoleptic and physicochemical properties. Anal. Chim. Acta.

[5] Singleton V.L., Rossi J.A. (1965). Colorimetry of total phenolics with phosphomolybdic-phosphotungstic acid reagents. Am. J. Enol. Vitic..

[6] Boulton R. (2001). The copigmentation of anthocyanins and its role in the color of red wine: A critical review. Am. J. Enol. Vitic..

[7] Xu Y., Simon J.E., Welch C., Wightman J.D., Ferruzzi M.G., Ho L., Passinetti G.M., Wu Q. (2011). Survey of polyphenol constituents in grapes and grape-derived products. J. Agric. Food Chem..

[8] Vinas P., Campillo N., Martínez-Castillo N., Hernández-Córdoba M. (2009). Solid-phase microextraction on-fiber derivatization for the analysis of some polyphenols in wine and grapes using gas chromatography–mass spectrometry. J. Chromatogr. A.

[9] Kontoudakis N., Esteruelas M., Fort F., Canals J.M., De Freitas V., Zamora F. (2011). Influence of the heterogeneity of grape phenolic maturity on wine composition and quality. Food Chem..

[10] Lorrain B., Ky I., Pechamat L., Teissedre P.L. (2013). Evolution of analysis of polyhenols from grapes, wines, and extracts. Molecules.

[11] Costa G., Noferini M., Fiori G., Torrigiani P. (2009). Use of Vis/NIR spectroscopy to assess fruit ripening stage and improve management in post-harvest chain. Fresh Prod..

[12] Tuccio L., Remorini D., Pinelli P., Fierini E., Tonutti P., Scalabrelli G., Agati G. (2011). Rapid and non-destructive method to assess in the vineyard grape berry anthocyanins under different seasonal and water conditions. Aust. J. Grape Wine Res..

[13] Giovenzana V., Beghi R., Malegori C., Civelli R., Guidetti R. (2014). Wavelength selection with a view to a simplified handheld optical system to estimate grape ripeness. Am. J. Enol. Vitic..

[14] Bramley R.G.V. (2005). Understanding variability in winegrape production systems 2. Within vineyard variation in quality over several vintages. Aust. J. Grape Wine Res..

[15] Cerovic Z.G., Moise N., Agati G., Latouche G., Ghozlen N.B., Meyer S. (2008). New portable optical sensors for the assessment of winegrape phenolic maturity based on berry fluorescence. J. Food Compos. Anal..

[16] Kolb C.A., Kopecký J., Riederer M., Pfündel E.E. (2003). UV screening by phenolics in berries of grapevine (*Vitis vinifera*). Funct. Plant Biol..

[17] Ben Ghozlen N., Cerovic Z.G., Germain C., Toutain S., Latouche G. (2010). Non-destructive optical monitoring of grape maturation by proximal sensing. Sensors.

[18] Kolb C.A., Pfündel E.E. (2005). Origins of non-linear and dissimilar relationships between epidermal UV absorbance and UV absorbance of extracted phenolics in leaves of grapevine and barley. Plant Cell Environ..

[19] Agati G., Meyer S., Matteini P., Cerovic Z.G. (2007). Assessment of anthocyanins in grape (*Vitis vinifera* L.) berries using a noninvasive chlorophyll fluorescence method. J. Agric. Food Chem..

[20] Baluja J., Diago M., Goovaerts P., Tardaguila J. (2012). Assessment of the spatial variability of anthocyanins in grapes using a fluorescence sensor: Relationships with vine vigour and yield. Precis. Agric..

[21] Savi S., Poni S., Moncalvo A., Frioni T., Rodschinka I., Arata L., Gatti M. (2019). Destructive and optical non-destructive grape ripening assessment: Agronomic comparison and cost-benefit analysis. PLoS ONE.

[22] Agati G., Cerovic Z.G., Pinelli P., Tattini M. (2011). Light-induced accumulation of ortho-dihydroxylated flavonoids as non-destructively monitored by chlorophyll fluorescence excitation techniques. Environ. Exp. Bot..

[23] Pedrós R., Goulas Y., Jacquemoud S., Louis J., Moya I. (2010). FluorMODleaf: A new leaf fluorescence emission model based on the PROSPECT model. Remote Sens. Environ..

[24] Ben Ghozlen N., Moise N., Latouche G., Martinon V., Mercier L., Besancon E., Cerovic Z. (2010). Assessment of grapevine maturity using a new portable sensor: Non-destructive quantification of anthocyanins. J. Int. Sci. Vigne Vin.

[25] Cerovic Z.G., Goutouly J.P., Hilbert G., Destrac-Irvine A., Martinon V., Moise N. (2009). Mapping winegrape quality attributes using portable fluorescence-based sensors. Frutic.

[26] Carreño J., Martínez A., Almela L., Fernández-López J. (1995). Proposal of an index for the objective evaluation of the colour of red table grapes. Food Res. Int..

[27] Fernández-López J.A., Almela L., Muñoz J.A., Hidalgo V., Carreño J. (1998). Dependence between colour and individual anthocyanin content in ripening grapes. Food Res. Int..

[28] Gambacorta G., Antonacci D., La Gatta M., Faccia M., La Gatta B., Pati S., Coletta A., La Notte E. (2011). Phenolic composition of Aglianico and Nero di Troia grapes and wines as affected by cover cropping and irrigation. Ital. J. Food Sci..

[29] Re R., Pellegrini N., Proteggente A., Pannala A., Yang M., Rice-Evans C. (1999). Antioxidant activity applying an improved ABTS radical cation decolorization assay. Free Radic. Biol. Med..

[30] Brand-Williams W., Cuvelier M.E., Berset C. (1995). Use of a free radical method to evaluate antioxidant activity. LWT Food Sci. Technol..

[31] Coletta A., Trani A., Faccia M., Punzi R., Dipalmo T., Crupi P., Antonacci D., Gambacorta G. (2013). Influence of viticultural practices and winemaking technologies on phenolic composition and sensory characteristics of Negroamaro red wines. Int. J. Food Sci..

[32] Revilla E., Ryan J.M. (2000). Analysis of several phenolic compounds with potential antioxidant properties in grape extracts and wines by high-performance liquid chromatography-photodiode array detection without sample preparation. J. Chromatogr. A.

[33] Brar H.S., Singh Z., Swinny E. (2008). Dynamics of anthocyanin and flavonol profiles in the ‘Crimson Seedless’ grape berry skin during development and ripening. Sci. Hortic..

[34] De la Cruz A.A., Hilbert G., Rivière C., Mengin V., Ollat N., Bordenave L., Decroocq S., Delaunay J.C., Delrot S., Mérillon J.M. (2012). Anthocyanin identification and composition of wild Vitis spp. accessions by using LC–MS and LC–NMR. Anal. Chim. Acta.

[35] Carreño J., Martínez A., Almela L., Fernández-López J.A. (1996). Measuring the color of table grapes. Color Res. Appl..

[36] Crupi P., Palattella D., Corbo F., Clodoveo M.L., Masi G., Caputo A.R., Battista F., Tarricone L. (2021). Effect of pre-harvest inactivated yeast treatment on the anthocyanin content and quality of table grapes. Food Chem..

[37] Brouillard R., Figueiredo P., Elhabiri M., Dangles O., Tomás-Barberán F.A., Robins R.J. (1997). Molecular interactions of phenolic compounds in relation to the colour of fruit and vegetables. Phytochemistry of Fruit and Vegetables.

[38] Pérez-Álvarez E.P., Molina D.I., Vivaldi G.A., García-Esparza M.J., Lizama V., Álvarez I. (2021). Effects of the irrigation regimes on grapevine cv. Bobal in a Mediterranean climate: I. Water relations, vine performance and grape composition. Agric Water Manag..

[39] Bergqvist J., Dokoozlian N., Ebisuda N. (2001). Sunlight exposure and temperature effects on berry growth and composition of Cabernet Sauvignon and Grenache in the Central San Joaquin Valley of California. Am. J. Enol. Vitic..

[40] Mori K., Sugaya S., Gemma H. (2005). Decreased anthocyanin biosynthesis in grape berries grown under elevated night temperature condition. Sci. Hortic..

[41] Fujita A., Soma N., Goto-Yamamoto N., Mizuno A., Kiso K., Hashizume K. (2007). Effect of shading on proanthocyanidin biosynthesis in grape berry. J. Jpn. Soc. Hort. Sci..

[42] De la Cerda-Carrasco A., López-Solís R., Nuñez-Kalasic H., Peña-Neira Á., Obreque-Slier E. (2015). Phenolic composition and antioxidant capacity of pomaces from four grape varieties (*Vitis vinifera* L.). J. Sci. Food Agric..

[43] Crupi P., Coletta A., Anna Milella R., Perniola R., Gasparro M., Genghi R., Antonacci D. (2012). HPLC-DAD-ESI-MS Analysis of Flavonoid Compounds in 5 Seedless Table Grapes Grown in Apulian Region. J. Food Sci..

[44] Vilanova M., Santalla M., Masa A. (2009). Environmental and genetic variation of phenolic compounds in grapes (*Vitis vinifera*) from northwest Spain. J. Agric. Sci..

[45] Hornedo-Ortega R., González-Centeno M.R., Chira K., Jourdes M., Teissedre P.L. (2020). Phenolic Compounds of Grapes and Wines: Key Compounds and Implications in Sensory Perception. Winemaking-Stabilization, Aging Chemistry and Biochemistry.

[46] Admane N., Genovese F., Altieri G., Tauriello A., Trani A., Gambacorta G., Verrastro V., Di Renzo G.C. (2018). Effect of ozone or carbon dioxide pre-treatment during long-term storage of organic table grapes with modified atmosphere packaging. LWT.

[47] Macheix J., Fleuriet A., Billot J. (1990). The main phenolics of fruits. Fruit Phenolics.

[48] Milella R.A., Antonacci D., Crupi P., Incampo F., Carrieri C., Semeraro N., Colucci M. (2012). Skin extracts from 2 Italian table grapes (Italia and Palieri) inhibit tissue factor expression by human blood mononuclear cells. J. Food Sci..

[49] Coletta A., Berto S., Crupi P., Cravero M.C., Tamborra P., Antonacci D., Daniele P.G., Prenesti E. (2014). Effect of viticulture practices on concentration of polyphenolic compounds and total antioxidant capacity of Southern Italy red wines. Food Chem..

[50] Heier A., Blaas W., Droß A., Wittkowski R. (2002). Anthocyanin analysis by HPLC/Esi-MS. Am. J. Enol. Vitic..

[51] Ryan J.M., Revilla E. (2003). Anthocyanin composition of Cabernet Sauvignon and Tempranillo grapes at different stages of ripening. J. Agric. Food Chem..

[52] Wang H., Race E.J., Shrikhande A.J. (2003). Anthocyanin transformation in Cabernet Sauvignon wine during aging. J. Agric. Food Chem..

[53] Liang Z., Owens C.L., Zhong G.Y., Cheng L. (2011). Polyphenolic profiles detected in the ripe berries of *Vitis vinifera* germplasm. Food Chem..

[54] Ferrara G., Mazzeo A., Matarrese A.M.S., Pacucci C., Punzi R., Faccia M., Trani A., Gambacorta G. (2015). Application of abscisic acid (S-ABA) and sucrose to improve colour, anthocyanin content and antioxidant activity of cv. Crimson Seedless grape berries. Aust. J. Grape Wine Res..

[55] Bahar A., Kaplunov T., Zutahy Y., Daus A., Lurie S., Lichter A. (2012). Auto-fluorescence for analysis of ripening in Thompson Seedless and colour in Crimson Seedless table grapes. Aust. J. Grape Wine Res..

[56] Bramley R., Le Moigne M., Evain S., Ouzman J., Florin L., Fadaili E., Hinze C., Cerovic Z. (2011). On-the-go sensing of grape berry anthocyanins during commercial harvest: Development and prospects. Aust. J. Grape Wine Res..

[57] Agati G., D’Onofrio C., Ducci E., Cuzzola A., Remorini D., Tuccio L., Lazzini F., Mattii G. (2013). Potential of a multiparametric optical sensor for determining in situ the maturity components of red and white *Vitis vinifera* wine grapes. J. Agric. Food Chem..

[58] Ferrandino A., Pagliarani C., Carlomagno A., Novello V., Schubert A., Agati G. (2017). Improved fluorescence-based evaluation of flavonoid in red and white winegrape cultivars. Aust. J. Grape Wine Res..

[59] Pinelli P., Romani A., Fierini E., Agati G. (2018). Prediction models for assessing anthocyanins in grape berries by fluorescence sensors: Dependence on cultivar, site and growing season. Food Chem..

[60] Ojeda H., Andary C., Kraeva E., Carbonneau A., Deloire A. (2002). Influence of pre-and postveraison water deficit on synthesis and concentration of skin phenolic compounds during berry growth of *Vitis vinifera* cv. Shiraz. Am. J. Enol. Vitic..

[61] Deluc L.G., Quilici D.R., Decendit A., Grimplet J., Wheatley M.D., Schlauch K.A., Mérillon J.M., Cushman J.C., Cramer G.R. (2009). Water deficit alters differentially metabolic pathways affecting important flavor and quality traits in grape berries of Cabernet Sauvignon and Chardonnay. BMC Genom..

