# Supplementary material for: Assessment of “Sugranineteen” Table Grape Maturation Using Destructive and Auto-Fluorescence Methods
Source: Foods. 2022 Feb 24;11(5):663. doi: 10.3390/foods11050663 (PMC8909905; doi:10.3390/foods11050663)
Supplement: Supplementary file 1 [file foods-11-00663-s001.zip › foods-1575441-supplementary.pdf]

Table S1. Relationships between the Multiplex® 3 ratios and the different quality parameters of Sugranineteen table grapes performed in this study. The ratios are described according to the manufacturer (Force A, Orsay, France).

**Ratio**	**Description**	**Formula**	**Correlation to [1]**	**Relationship with [2]**	**n ***	**R-sq**	***Significance***
ANTH_RG	Log of FER_RG	log (FRF_R/FRF_G)	Anthocyanins	Anthocyanins	16	0.9613	*$p \leq 0.001$, ****
				CIRG	7	0.8713	*$p = 0.02$, **
FERARI	Log of FRF_R	Log (5000/FRF_R)	Anthocyanins	Anthocyanins	16	0.8743	*$p \leq 0.001$, ****
FLAV	Flavonols	log (FRF_R/FRF_UV)	Flavonols	Flavonoids	15	nd	*ns*
SFR_R	Simple Fluorescence Ratio	FRF_R / RF_R	Chlorophyll	TSS	9	0.7954	*$p = 0.001$, ****
				TA	9	0.6186	*$p = 0.012$, ***
				CIRG	10	0.7835	*$p = 0.005$, ***
NBI_R	Nitrogen Balance Index	FRF_UV / RF_R	Epidermal phenolics and chlorophyll	Anthocyanins	16	0.8032	*$p \leq 0.001$, ****
				Flavonoids	9	0.4773	*$p = 0.039$, **

[1]: According to the user guide of Multiplex® 3 and previous studies [17–55], the mentioned ratios are correlated to the corresponding above-mentioned parameters. [2]: Regression equations investigated in this study. n, sample size; R-sq, coefficient of determination (R^2); nd, not detected; *ns*, not significant; TSS, total soluble solids, TA, titratable acidity; CIRG, color index of red grapes. * Each n represents the mean value of triplicates of laboratory analysis and the relative 196 in–vineyard collected fluorescent measurements.
